# Remofuscin induces xenobiotic detoxification via a lysosome-to-nucleus signaling pathway to extend the *Caenorhabditis elegans* lifespan

**DOI:** 10.1038/s41598-022-11325-2

**Published:** 2022-05-03

**Authors:** Miae Oh, Jiah Yeom, Ulrich Schraermeyer, Sylvie Julien-Schraermeyer, Young-Hee Lim

**Affiliations:** 1 Department of Integrated Biomedical and Life Science, Graduate School, Korea University, Seoul, 02841 Republic of Korea; 2 Division of Experimental Vitreoretinal Surgery, Centre for Ophthalmology, University of Tübingen, 72076 Tübingen, Germany; 3 School of Biosystems and Biomedical Sciences, Korea University, Seoul, 02841 Republic of Korea; 4 Department of Laboratory Medicine, Korea University Guro Hospital, Seoul, 08308 Republic of Korea

**Keywords:** Mechanism of action, Ageing

## Abstract

Lipofuscin is a representative biomarker of aging that is generated naturally over time. Remofuscin (soraprazan) improves age-related eye diseases by removing lipofuscin from retinal pigment epithelium (RPE) cells. In this study, the effect of remofuscin on longevity in *Caenorhabditis elegans* and the underlying mechanism were investigated. The results showed that remofuscin significantly (*p* < 0.05) extended the lifespan of *C. elegans* (N2) compared with the negative control. Aging biomarkers were improved in remofuscin-treated worms. The expression levels of genes related to lysosomes (*lipl-1* and *lbp-8*), a nuclear hormone receptor (*nhr-234*), fatty acid beta-oxidation (*ech-9*), and xenobiotic detoxification (*cyp-34A1, cyp-35A1, cyp-35A2, cyp-35A3, cyp-35A4, cyp-35A5, cyp-35C1, gst-28,* and *gst-5*) were increased in remofuscin-treated worms. Moreover, remofuscin failed to extend the lives of *C. elegans* with loss-of-function mutations (*lipl-1, lbp-8, nhr-234, nhr-49, nhr-8, cyp-35A1, cyp-35A2, cyp-35A3, cyp-35A5,* and *gst-5*), suggesting that these genes are associated with lifespan extension in remofuscin-treated *C. elegans*. In conclusion, remofuscin activates the lysosome-to-nucleus pathway in *C. elegans*, thereby increasing the expression levels of xenobiotic detoxification genes resulted in extending their lifespan.

## Introduction

Aging induces metabolic changes such as lipofuscin accumulation, which thus serve as a biomarker of aging^1^. The accumulation of lipofuscin that is composed of highly oxidized proteins, lipids, and metals, is enhanced by ROS produced by damaged mitochondria and lysosomes and causes eye diseases and neurodegenerative diseases^2^. Remofuscin (soraprazan, (7*R*,8*R*,9*R*)-7-(2-methoxyethoxy)-2,3-dimethyl-9-phenyl-7,8,9,10-tetrahydroimidazo[1,2-h][1,7]naphthyridin-8-ol) was developed to treat age-related macular degeneration (AMD) and Stargardt disease by removing lipofuscin from retinal pigment epithelium (RPE) cells. AMD and Stargardt disease are induced by aging or an innate defect in ATP binding cassette subfamily A member 4 (ABCA4), and lipofuscin accumulates in RPE cells, which may cause blindness^3,4^. The produced lipofuscin is not easily degraded or excreted out of the aged cell^5^, but remofuscin was shown to remove significant amounts of lipofuscin from RPE cells in cynomolgus monkeys^6^.

The *C. elegans* model is suitable for studying various biological functions, including human diseases and aging^7^. *C. elegans* grows on agar plates and eats microbes and is therefore easy and cheap to manipulate in the laboratory. The *C. elegans* body is transparent and visible with a microscope. *C. elegans*, which propagates mainly through self-fertilization, has a particularly short lifespan, and its whole genome has been sequenced. Moreover, approximately half of *C. elegans* genes have human orthologs, and approximately 70% of genes related to human diseases have homologs in *C. elegans*^8^. As *C. elegans* ages, autofluorescent lipofuscin accumulates in the intestine. Thus, *C. elegans* is a good model to study lifespan extension by decreasing lipofuscin accumulation because lipofuscin accumulation shortens the *C. elegans* lifespan.

Lifespan extension derives from many factors, such as diet restriction (DR) and strengthened immunity, in a wide range of taxa ranging from yeast to primates^9,10^. Many of the pathways regulating lifespan are linked to lipid metabolism, and lipids act as signaling molecules in longevity signaling pathways. Lipid metabolism is also altered over time, and aging and longevity are thus regulated by lipid signaling^11^. Beta-oxidation genes are upregulated in a diet restriction (DR)-like state, consequently reducing the amount of stored fat, which leads to lower reactive oxygen species (ROS) levels. ROS are related to aging and oxidative damage^12^. DR is one of the most influential environmental interventions that extends the lifespans of a variety of species^13^. Increased fatty acid beta-oxidation induces the expression of xenobiotic detoxification genes to clear lipophilic endotoxins produced during lipid catabolism, and the resulting metabolic shift increases the longevity of *C. elegans*^14^. In the DR state, lysosomes play an important role in the early catabolic steps of lipid degradation^15^. *C. elegans* has eight lysosomal lipases, LIPL-1 to LIPL-8^16^; LIPL-4 has been extensively studied because it plays important roles in autophagy, fat metabolism, and lysosomal activity, which are linked to longevity in *C. elegans*^17–19^. Among the lysosomal lipase genes, *lipl-1* is the most upregulated in the fasting state, and its sequence is similar to that of human lysosomal acid lipase (BLAST scores 9e−78)^20^. However, the mechanism by which LIPL-1 extends the lifespan of *C. elegans* has not been studied.

In this study, we hypothesized that remofuscin would extend the lifespan possibly by preventing lipofuscin accumulation because lipofuscin accumulates over time and remofuscin prevents lipofuscin accumulation. Therefore, we herein investigated the effect of remofuscin on lifespan extension in *C. elegans* and elucidated the underlying mechanism.

## Results

### Remofuscin extends the lifespan of *C. elegans*

Remofuscin significantly (*p* < 0.05) increased the mean lifespan (MLS) of wild-type *C. elegans* (N2) in a dose-dependent manner compared with that of the NC (negative control, 0 μM remofuscin) (Table 1, Supplementary data). Compared with those of the NCs, the MLSs of *C. elegans* (N2) treated with 50 µM, 100 µM, and 200 µM remofuscin were increased by 9.9%, 14.6%, and 20.4%, respectively. The survival percentages of the worms treated with remofuscin were higher than those of the untreated worms after 5 days (Supplementary Fig. S1).

**Table 1:** The mean lifespans (MLSs) of *C. elegans* (N2) treated with various concentrations of remofuscin.

Remofuscin (µM)	No. of total worms (dead/censored)	MLS ± SE (days)	MLS change (%, relative to the NC)
0	135 (116/19)	12.11 ± 0.70	-
50	135 (112/23)	13.31 ± 0.72*	9.9
100	135 (117/18)	13.88 ± 0.75***	14.6
200	135 (111/24)	14.58 ± 0.80***	20.4

All experiments were conducted three times independently. *p < 0.05, ***p < 0.001, log-rank test, compared with the NC (0 μM remofuscin).

### Effects of remofuscin on age-related factors in *C. elegans*

**Figure 1:**
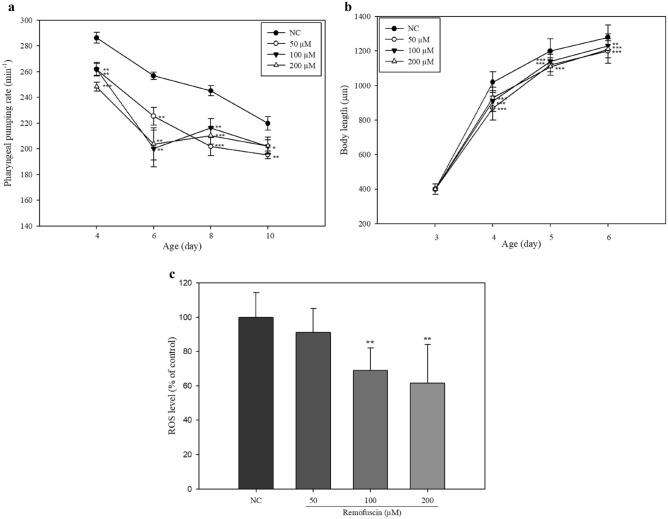
Effect of remofuscin on pharyngeal pumping rate, body size, and ROS generation in *C. elegans* (N2). Three-day-old (day 1 of the adult stage) *C. elegans* fed on an *E. coli* OP50 lawn were transferred onto fresh mNGM plates containing *E. coli* OP50 and various concentrations of remofuscin. The pumping rate in the terminal bulb was measured for 1 min every 48 h from day 4 to day 10, and the mean rate of 15 worms from each group was determined (**A**). The body lengths of 10 worms from each group were measured (**B**). ROS levels in remofuscin-treated *C. elegans* (N2) (**C**). The relative ROS levels were measured after 14 days of treatment with remofuscin. The fluorescence signal in each group (more than 80 worms for each measurement) was normalized to the protein concentration in the group. All experiments were conducted three times independently. ^*^p < 0.05, ^**^p < 0.01, ^***^p < 0.001, Student’s t-test, compared with the NC (0 μM remofuscin).

To investigate the effect of remofuscin on lifespan extension in *C. elegans*, pharyngeal pumping rate, an age-related biomarker was measured. A reduced pharyngeal pumping rate indicates a decrease in feeding, which can induce a DR-like state^21^. DR reduces the body size of *Caenorhabditis elegans*, which might be associated with an extended lifespan^22^. The pharyngeal pumping rates of the remofuscin-treated groups were significantly (*p* < 0.05) decreased compared with that of the NC group (Fig. 1A). Regardless of whether *C. elegans* was treated with or without remofuscin, the pharyngeal pumping rate decreased until day 6; however, the pumping rates of worms treated with 100 μM and 200 μM remofuscin on day 8 and day 10 were nearly similar or even higher than those on day 6. In contrast, the pharyngeal pumping rate of the NC group continuously decreased over 10 days. The body lengths of the live worms were measured every 24 h until 6 days of age. While the body length increased with age in all the groups, those of the remofuscin-treated groups were significantly shorter than those of the NC group (Fig. 1B). The results suggest that the decrease of pharyngeal pumping rate in remofuscine-treated *C. elegans* might induce DR-like state, consequently reduce the body size of *C. elegans*.

To investigate the inhibitory effect of remofuscin on ROS generation in *C. elegans*, the total ROS levels in worms treated with remofuscin were measured. The ROS levels in *C. elegans* grown on plates containing 100 μM and 200 μM remofuscin for 14 days were significantly (*p* < 0.05) decreased compared with those in the NC group (Fig. 1C). The results suggest that remofuscin-induced ROS reductions might affect lifespan extension in *C. elegans*.

**Figure 2:**
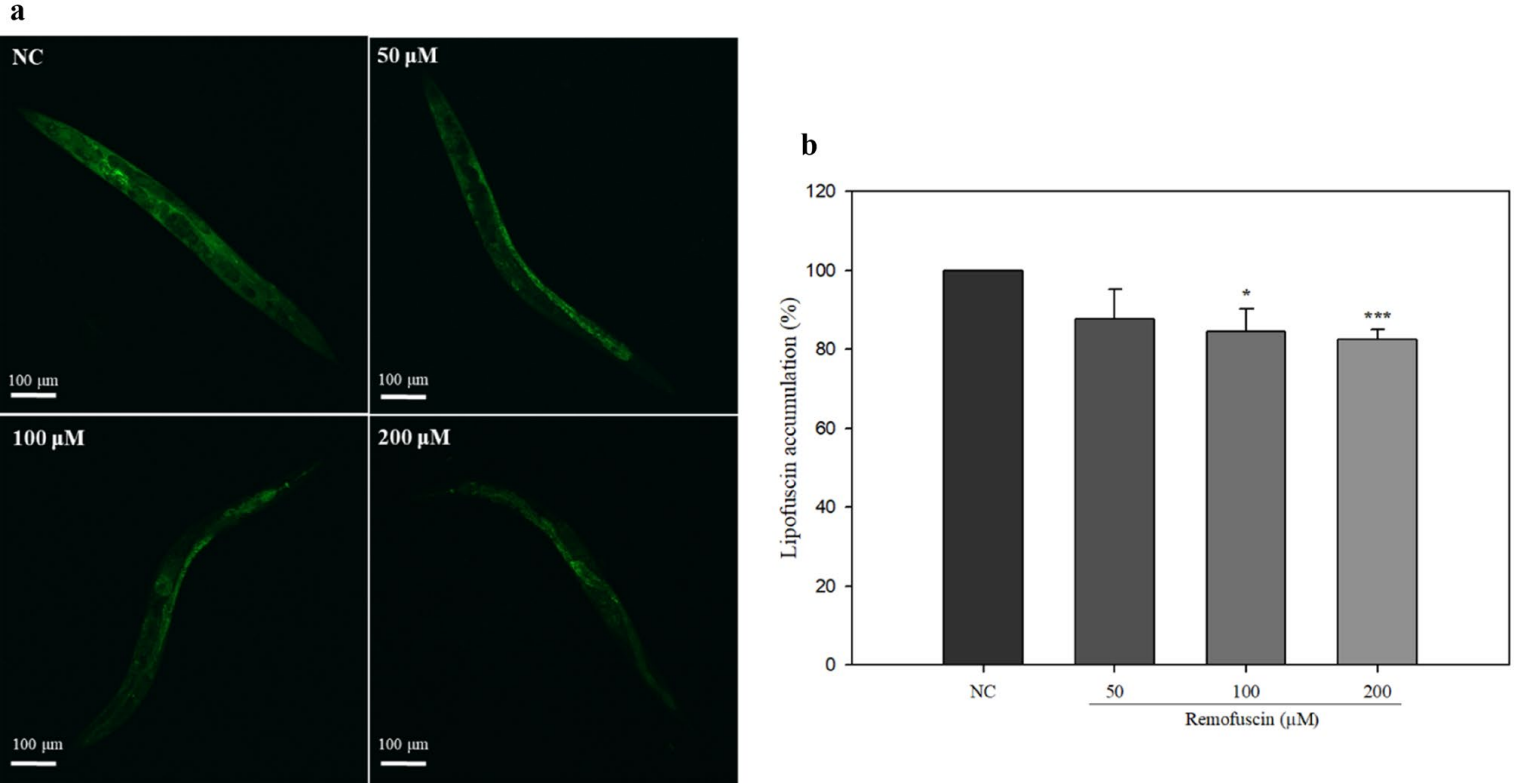
Accumulation of lipofuscin in remofuscin-treated *C. elegans* (N2). (**A**) Images of lipofuscin fluorescence in worms treated with various concentrations of remofuscin on day 14 (scale bar = 100 µm). (**B**) The lipofuscin fluorescence in the worms was quantified using ImageJ software. Ten worms from each group were used for the measurements. All experiments were conducted three times independently. ^*^p < 0.05, ^***^p < 0.001, Student’s t-test, compared with the NC (0 μM remofuscin).

Lipofuscin accumulation serves as a biomarker of aging, and remofuscin significantly decreased the lipofuscin levels in *C. elegans* treated with 100 μM and 200 μM remofuscin on day 14 (Fig. 2). These results suggest that remofuscin affects the lifespan of *C. elegans*.

### Microarray analysis of remofuscin-treated *C. elegans*

**Figure 3:**
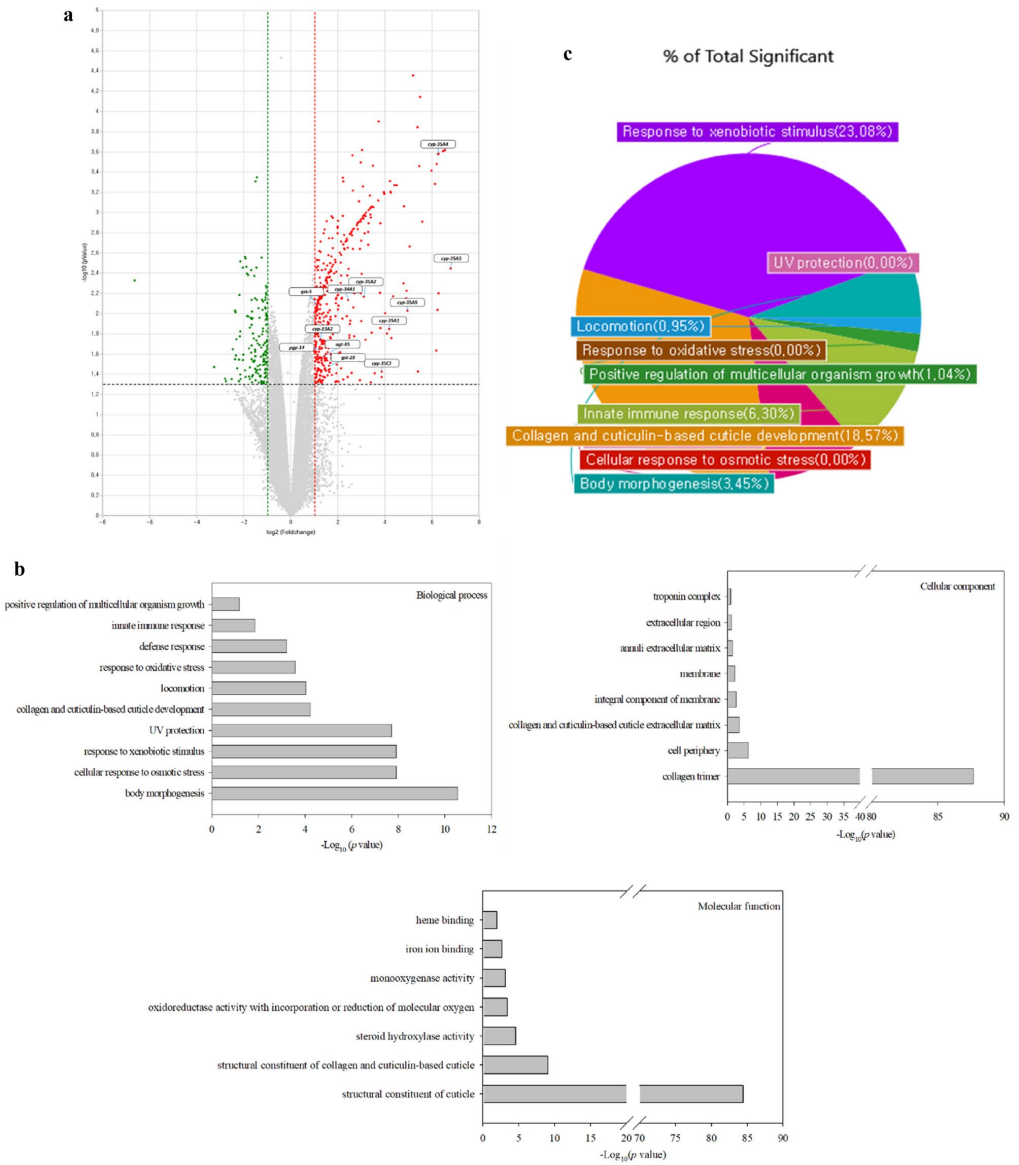
Differential gene expression between the negative control and 200 µM remofuscin-treated groups as determined by RNA microarray analysis. DEG analysis software (ExDEGA v.1.6.8, Ebiogen, KOR) was used to display the gene expression data as a volcano plot (**A**). The genes in the boxes are related to xenobiotic detoxification shown in Supplementary Table S1 (fold change ≥ 2.0 and *p* < 0.05). The DAVID functional annotation tool was used to analyze the gene ontology terms (**B**), and the biological processes associated with the DEGs revealed by microarray analysis are shown as a pie chart (**C**).

To investigate the mechanism by which remofuscin promotes longevity, microarray analysis of wild-type (N2) worms treated with 0 µM (NC) and 200 µM remofuscin for 14 days was performed. A total of 31,383 genes were identified, among which 340 and 103 genes were significantly (*p* < 0.05) upregulated (≥ twofold) and downregulated (≤ 0.5-fold), respectively, between the NC and 200 µM remofuscin-treated worms (Fig. 3A). Based on the terms represented in the GO database, the differentially expressed genes (DEGs) were divided into three categories: biological process (BP), cellular component (CC), and molecular function (MF). GO analysis showed 10 major functional categories in the BP group, 8 categories in the CC group, and 7 categories in the MF group based on the criterion for identifying differentially expressed genes by microarray analysis (fold change ≥ 2.0 and *p* < 0.05) (Fig. 3B). Among these genes, those related to response to xenobiotic stimuli were highly expressed in the 200 μM remofuscin-treated worms compared with the NC worms. Xenobiotic detoxification is known to affect longevity in *C. elegans,* and based on the total significance (Fig. 3C), we focused on the genes related to xenobiotic stimulus responses. The genes related to xenobiotic metabolism (GO: 0006805) (*cyp-13A2*, *cyp-34A1*, *cyp-35A1*, *cyp-35A2*, *cyp-35A3*, *cyp-35A4*, *cyp-35A5*, *cyp-35C1*, *gst-28*, *gst-5*, *ugt-65*, *pgp-14*, and *folt-2*) were significantly (*p* < 0.05) increased (> twofold) compared with those in the NC group (0 µM remofuscin) as determined by microarray analysis (Supplementary Table S1).

### Effects of remofuscin on the expression levels of genes related to lysosomes and xenobiotic metabolism

**Figure 4:**
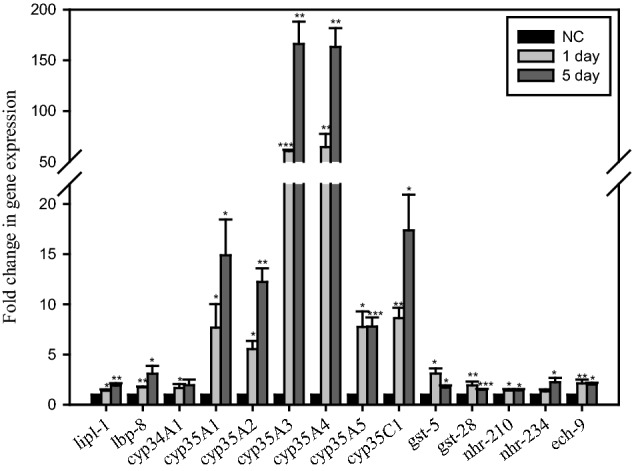
Expression levels of genes related to xenobiotic metabolism, lysosomes, and NHRs in remofuscin-treated *C. elegans*. Worms were grown on NGM plates containing 0 µM and 200 µM remofuscin for 1 and 5 days, and the gene expression levels were measured by qPCR. The experiments were independently conducted at least three times. ^*^p < 0.05, ^**^p < 0.01, ^***^p < 0.001, Student’s t-test, compared with the NC (0 μM remofuscin) on each day.

Based on the microarray results, the expression levels of genes related to lysosomal lipases (*lipl-1*), long-chain fatty acid transporters (*lbp-8*), fatty acid beta-oxidation (*ech-9*), and xenobiotic detoxification (*cyp-35A* subfamily, *gst-5*, and *gst-28*) in *C. elegans* (N2) treated with 0 and 200 µM remofuscin for 1 day and 5 days were measured by qPCR. The expression levels of genes related to xenobiotic metabolism, especially *cyp35A* subfamily genes, were significantly increased in remofuscin-treated worms compared with NC worms (Fig. 4). Mostly, the gene expression levels were higher on day 5 than on day 1. In addition, the expression levels of *lipl-1*, *lbp-8*, and *ech-9* were significantly increased in remofuscin-treated worms. Although the differences were not statistically significant, the levels of several nuclear hormone receptor (NHR) genes, transcription factors of cytochrome p450 family genes, were increased by more than twofold as determined by microarray analysis (Supplementary Table S2). Among them, the levels of only *nhr-210* and *nhr-234* were significantly increased in remofuscin-treated worms compared with the NC worms as determined by qPCR analysis (Fig. 4).

### Longevity assay of loss-of-function mutants

To elucidate the mechanism by which remofuscin extends the *C. elegans* lifespan, loss-of function mutants for genes related to xenobiotic detoxification were used for longevity assays. The loss-of function mutants available from the CGC and NBRP were selected based on the qPCR results. In addition, although the expression levels of the transcription factor genes (*nhr-49*, *nhr-8*, *ahr-1*, and *pha-4*) related to lipid metabolism and xenobiotic stimulus responses in *C. elegans* were not upregulated in remofuscin-treated worms compared with the NC (Supplementary Fig. S2), loss-of function mutants were used to assess lifespan extension based on previous reports that they regulate the expression of genes related to lipid and xenobiotic metabolism^14,23–26^. Remofuscin treatment failed to extend the lifespans of worms exhibiting loss-of-function mutations of genes related to lipid metabolism (*lipl-1* and *lbp-8*) and xenobiotic detoxification (*cyp-35A1, cyp-35A2, cyp-35A3, cyp-35A5,* and *gst-5*) (Table 2, Supplementary data, Supplementary Fig. S3). Interestingly, remofuscin failed to extend the lifespan of *C. elegans* harboring transcription factor (*nhr-49*, *nhr-8*, and *nhr-234*) mutants but not that of worms with the *nhr-210* mutant. Although the *ahr-1* and *pha-4* deletion extended the lifespans of 100 μM and 200 μM remofuscin-treated worms, respectively, their overall MLSs were decreased by remofuscin treatment compared with the wild type.

**Table 2:** The mean lifespans of *C. elegans* with loss-of-function mutants.

Nematode type	Remofuscin (µM)	MLS ± SE (days) (n)	MLS change (%, relative to the NC)
FX1954 *lipl-1* (tm1954)	0	10.03 ± 0.60 (107)	–
	50	10.35 ± 0.62 (113)	3.19
	100	10.12 ± 0.67 (101)	0.90
	200	10.30 ± 0.64 (115)	2.69
VC4077 *lbp-8* (gk5151)	0	11.12 ± 0.56 (125)	–
	50	11.32 ± 0.57 (123)	1.80
	100	11.13 ± 0.62 (122)	0.09
	200	11.34 ± 0.64 (114)	1.98
FX1290 *nhr-210* (tm1290)	0	10.43 ± 0.57 (117)	–
	50	10.71 ± 0.64 (113)	2.68
	100	11.36 ± 0.68** (109)	8.92
	200	11.78 ± 0.70*** (112)	12.94
VC1806 *nhr-234* (gk865)	0	10.55 ± 0.58 (120)	–
	50	10.54 ± 0.63 (114)	− 0.09
	100	10.65 ± 0.61 (119)	0.95
	200	10.51 ± 0.62 (116)	− 0.38
FX30306 *nhr-49* (tm7967)	0	5.83 ± 0.33 (109)	–
	50	5.71 ± 0.32 (109)	− 2.06
	100	5.80 ± 0.31 (111)	− 0.51
	200	6.03 ± 0.34 (115)	3.43
FX19275 *nhr-8* (tm1800)	0	10.09 ± 0.53 (117)	–
	50	10.34 ± 0.58 (104)	2.48
	100	10.25 ± 0.57 (107)	1.59
	200	10.02 ± 0.56 (110)	− 0.69
FX01722 *ahr-1* (tm1722)	0	11.00 ± 0.66 (109)	–
	50	11.08 ± 0.66 (107)	0.73
	100	11.61 ± 0.69* (106)	5.55
	200	11.37 ± 0.67 (113)	3.36
FX4598 *pha-4* (tm4598)	0	9.29 ± 0.62 (109)	–
	50	10.04 ± 0.61 (114)	8.07
	100	10.46 ± 0.56 (120)	12.59
	200	10.53 ± 0.59* (118)	13.35
VC875 *cyp-35A1* (ok1414)	0	7.58 ± 0.44 (101)	–
	50	8.09 ± 0.46 (100)	6.73
	100	7.51 ± 0.47 (100)	− 0.92
	200	7.29 ± 0.40 (106)	− 3.83
FX21842 *cyp-35A2* (tm11844)	0	9.68 ± 0.58 (107)	–
	50	10.11 ± 0.59 (113)	4.44
	100	10.20 ± 0.62 (111)	5.37
	200	10.34 ± 0.61 (113)	6.82
RB2046 *cyp-35A3* (ok2709)	0	9.41 ± 0.64 (117)	–
	50	9.26 ± 0.63 (113)	− 1.59
	100	9.20 ± 0.61 (125)	− 2.23
	200	9.64 ± 0.65 (117)	2.44
FX22344 *cyp-35A5* (tm12345)	0	8.38 ± 0.61 (112)	–
	50	8.45 ± 0.57 (120)	0.84
	100	8.45 ± 0.61 (120)	0.84
	200	8.71 ± 0.60 (111)	3.94
RB2063 *gst-5* (ok2726)	0	7.36 ± 0.48 (110)	–
	50	7.38 ± 0.48 (112)	0.27
	100	7.81 ± 0.50 (118)	6.11
	200	7.76 ± 0.50 (114)	5.43

All experiments were conducted three times independently. n, The number of total worms; *p < 0.05, **p < 0.01, ***p < 0.001, log-rank test, compared with the NC (0 μM remofuscin).

### Role of NHR-234 in the longevity mechanism of remofuscin

**Figure 5:**
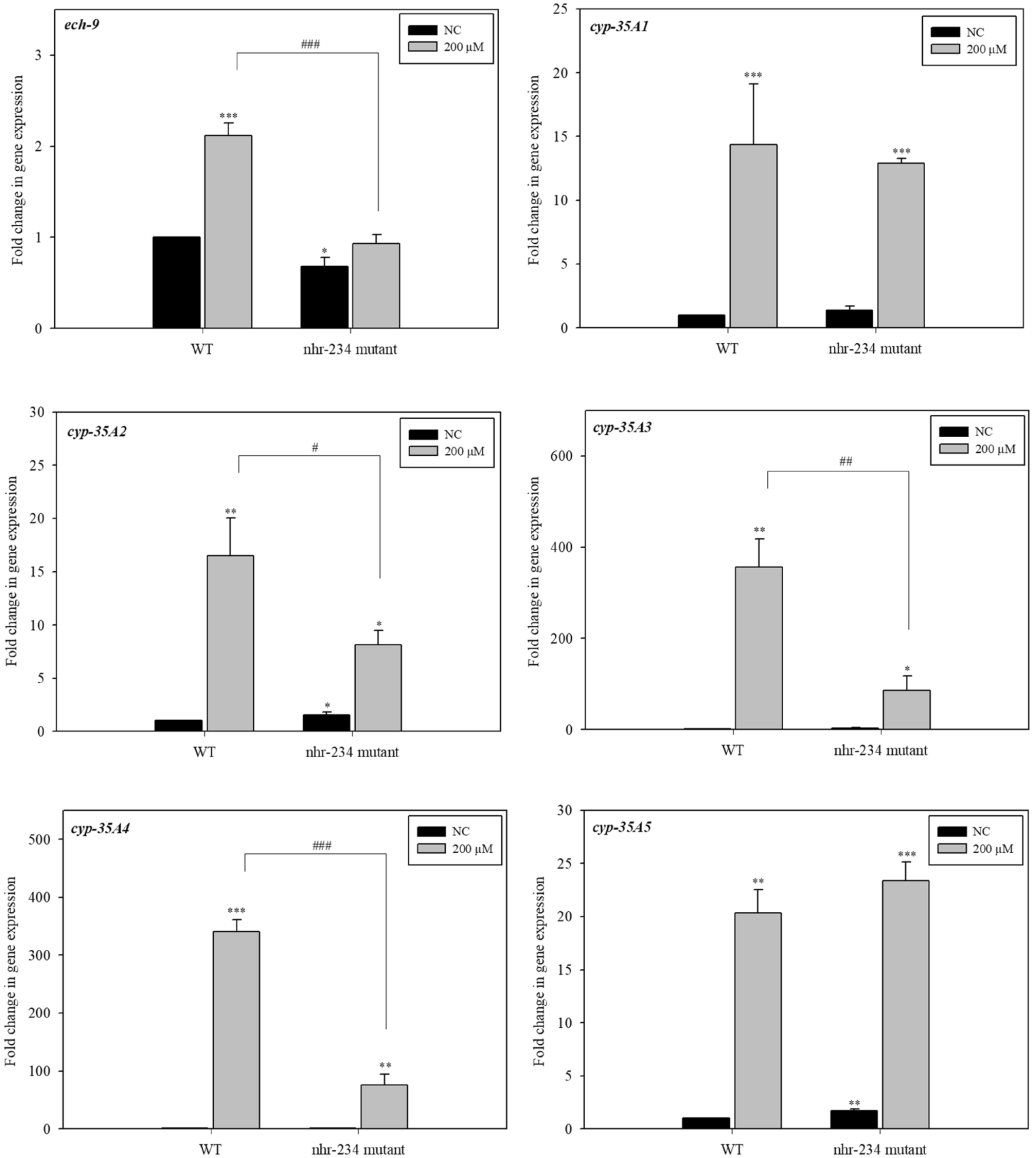
Expression levels of *ech-9* and *cyp-35A* subfamily members in wild-type (N2) (WT) and *nhr-234* mutant *C. elegans*. The worms were grown on NGM plates containing 0 µM and 200 µM remofuscin for 5 days, and the gene expression levels were measured by qPCR. The experiments were independently conducted at least three times. ^*^p < 0.05, ^**^p < 0.01, ^***^p < 0.001, Student’s t-test, compared with the wild-type (N2) NC (0 μM remofuscin) and ^###^*p* < 0.001, Student’s t-test, compared with the 200 µM remofuscin-treated wild-type (N2) worms.

NHR-234 cooperates with NHR-49 to induce the transcription of target genes related to lipid metabolism. Remofuscin-treated nematodes upregulated *nhr-234* expression compared with that in the control group, and the *nhr-234* mutant failed to extend the lifespan of the worms. To investigate the effect of NHR-234 on the expression of genes related to lipid metabolism and xenobiotic detoxification proposed to be downstream of NHR-234 in *C. elegans* treated with remofuscin, the expression levels of *ech-9*, *cyp-35A1*, *cyp-35A2*, *cyp-35A3*, *cyp-35-A4*, *and cyp-35A5* in the *nhr-234* deletion mutants were analyzed by qPCR. The expression levels of *ech-9*, *cyp-35A2*, *cyp-35A3*, and *cyp-35-A4*, but not *cyp-35A1* and *cyp-35A5*, were not increased in the *nhr-234* deletion mutants treated with remofuscin (200 µM) compared with the wild-type (N2) worms, indicating that NHR-234 might regulate their expression (Fig. 5).

## Discussion

Remofuscin reduced the lipofuscin accumulation in the RPE cells of cynomolgus monkeys^6^. In this study, we found that remofuscin reduced the lipofuscin accumulation in *C. elegans*, which was potentially related to the significant MLS extension in remofuscin-treated *C. elegans*. In *C. elegans*, despite an abundant supply of food, a reduced pharyngeal pumping rate is associated with a lower food intake, which might induce a DR-like state in which energy metabolism shifts from glucose to lipid oxidation, resulting in the upregulation of beta-oxidation genes^27^. The generation of ROS via beta-oxidation is reduced in lipid catabolism compared with glucose catabolism^14^. Herein, remofuscin reduced the pharyngeal pumping rate and consequently reduced the production of ROS, which may have contributed to the lifespan extension of remofuscin-treated *C. elegans*. In the DR state, the lipid catabolism rate increases to supply energy, and lipid catabolism generates lipid metabolites that may mediate lipotoxicity^14^. The microarray and qPCR analyses of remofuscin-treated worms in a DR-like state revealed that genes related to lysosomes (*lipl-1* and *lbp-8*), beta-oxidation (*ech-9*), and xenobiotic detoxification (*cyp-34A1*, *cyp-35A1*, *cyp-35A2*, *cyp-35A3*, *cyp-35A4*, *cyp-35A5*, *cyp-35C1*, *gst-28*, and *gst-5*) were upregulated, which may have prevented damage caused by lipid metabolites.

*C. elegans* has 8 lysosomal lipases, LIPL-1 to LIPL-8, among which LIPL-4 plays a role in the lifespan extension of *C. elegans*^24^. Overexpression of LIPL-4 induces the expression of the lipid chaperone protein LBP-8, and mitochondrial beta-oxidation is activated via NHR-49 and NHR-80 to promote lipid catabolism. ROS in mitochondria (mtROS) activate the transcription factor JUN-1, and the LIPL-4 and LBP-8 signaling pathways induce antioxidant targets and oxidative stress tolerance, thereby extending the *C. elegans* lifespan^26^. However, remofuscin did not increase the expression level of *jun-1* in *C. elegans*. The *C. elegans* genes directly or indirectly involved in lipid catabolism, especially LIPL-1, respond to the DR state and control lipid metabolism^20^. In this study, the expression levels of genes related to a lysosomal lipase (*lipl-1*), a lipid binding protein (*lbp-8*), and beta-oxidation (*ech-9*) were increased in remofuscin-treated worms. In addition to *jun-1*, the transcription factors *nhr-49* and *nhr-27* play roles in the longevity of *C. elegans* by reducing the mitochondrial electron transport chain^28^. Unlike mammals, which have only 48 NHR genes, *C. elegans* has 284 NHR genes that play roles in various processes, including lipid and xenobiotic metabolism. In particular, NHR-49*,* a homolog of mammalian hepatocyte nuclear factor 4 (HNF4) that functions similarly to peroxisome proliferator-activated receptors (PPARs), is known to transcriptionally regulate many genes related to beta-oxidation, including acyl-CoA synthetase, enoyl-CoA hydratase, and carnitine palmitoyl transferase^29,30^. However, the effect of NHR-49 on *ech-9* (enoyl-CoA hydratase gene) remains debatable^14,29^. In this study, although the expression level of the *nhr-49* gene was not increased, that of *ech-9* was increased in *C. elegans* treated with remofuscin. In addition, the *nhr-49* deletion failed to extend the lifespan of remofuscin-treated worms, indicating the possible role of NHR-49 in the longevity of remofuscin-treated *C. elegans*. Interestingly, we found that the gene expression of nuclear receptor NHR-234, which is known to cooperate with NHR-49, was significantly upregulated in remofuscin-treated *C. elegans* and that the *nhr-234* mutant failed to extend the lifespan of the worms. Information on the function of *nhr-234* in *C. elegans* is limited. Unlike the wild-type (N2) worms, those with *nhr-234* deletion mutants (VC1806) did not exhibit increased expression levels of the genes related to lipid metabolism (*ech-9*) and xenobiotic stimulus responses (*cyp-35A2, cyp-35A3,* and *cyp-35A4*), which may be regulated by NHR-234. Thus, NHR-234 with or without NHR-49 plays a role in the longevity of remofuscin-treated worms by regulating *ech-9* expression and thereafter altering the expression of genes related to xenobiotic detoxification.

Endogenous lipofuscin, the lipid-containing product resulting from the oxidation of unsaturated fatty acids composed of digested lipid-containing lysosomal residues, accumulates over time and can be a xenobiotic. In addition, remofuscin may act as an exogenous xenobiotic agent. Dependence on fatty acid oxidation for energy sources leads to the formation of lipophilic endotoxins and, in turn, activates xenobiotic detoxification genes^31,32^. Xenobiotic detoxification occurs in three phases: phase I (cytochrome p450 enzymes (CYPs) chemically modify endotoxins), phase II (UDP-glucuronosyl transferases and glutathione S-transferases (GSTs) make them more soluble), and finally, phase III (modified endotoxins are emitted into the extracellular space by ATP-binding cassette transporters)^32,33^. In this study, the expression levels of *cyp* (cytochrome p450 enzymes) and *gst* (glutathione S-transferases) genes were increased in remofuscin-treated *C. elegans*. GST functions as an antioxidant in detoxification reactions and inhibits ROS generation. In addition, GST stops or slows lipofuscin formation and cleaves the existing lipofuscin^34^. The transcription factors NHR-8, AHR-1, and PHA-4 regulate the expression of genes related to xenobiotic metabolism. NHR-8 is required for xenobiotic resistance and may regulate the expression of cytochrome P450 genes in *C. elegans*^23^, AHR-1 binding to the xenobiotic response element (XRE), which is related to CYP-35A subfamily members possessing XRE like elements in the promoter regions, regulates lipid signaling^35^, and PHA-4 induces the expression of xenobiotic detoxification genes^14^. In this study, we found that *nhr-8* deletion failed to extend the lifespan of remofuscin-treated worms, which means that genes related to xenobiotic detoxification activated by remofuscin conferred *C. elegans* with longevity.

**Figure 6:**
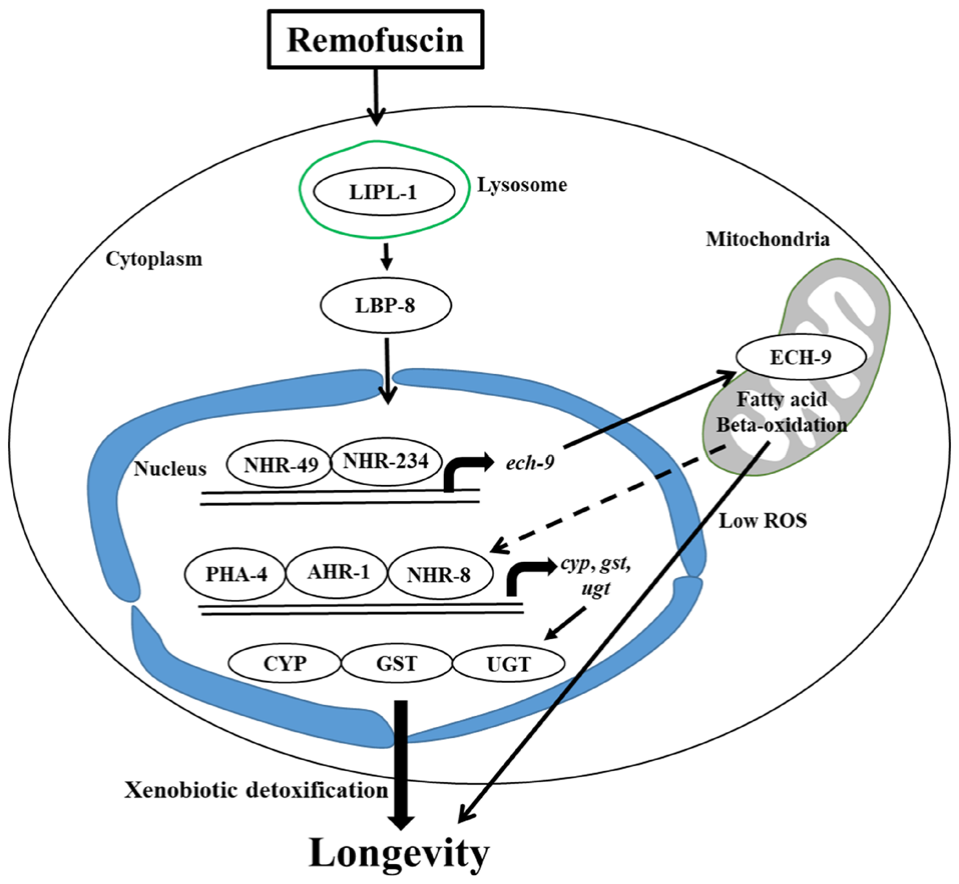
Predicted mechanism by which remofuscin extends the *C. elegans* lifespan. A lysosomal lipase, LIPL-1, activates lipid catabolism, similar to that in a diet restriction (DR)-like state, thereby decreasing the ROS levels and subsequently activating the xenobiotic detoxification process. This sequence ultimately extends the lifespan of remofuscin-treated *C. elegans*. The broken line shows the predicted route based on the previously reported pathway^14^.

Based on the results obtained in this study, we predicted a pathway that is associated with lifespan extension in remofuscin-treated *C. elegans* (Fig. 6). Remofuscin increases the expression of lysosomal lipase and induces lipid catabolism (beta-oxidation), subsequently activating the xenobiotic detoxification response and extending the *C. elegans* lifespan. In addition, remofuscin-treated worms enter a DR-like state by decreasing their pharyngeal pumping rate, which is followed by a reduction in ROS levels through fatty acid beta-oxidation, thereby contributing to their lifespan extension. Lysosomal signaling from LIPL-4 to LBP-8 followed by NHR-49 and NHR-80 promotes the longevity of *C. elegans*^36^; however, the signal herein was observed from LIPL-1 to LBP-8, followed by NHR-234 and/or NHR-49.

Remofuscin is also known as a potent and reversible inhibitor of the $\small {H}^{+}/{K}^{+}$ ATPase proton pump in cynomolgus monkeys^6^. Although the proton pump which is inhibited by remofuscin is not present in the RPE in the eyes of human, proton pump inhibitors are known to increase the lysosomal pH, thereby activating transcription factor EB (TFEB), a master transcriptional regulator of lysosomal biogenesis, and inducing lysosomal exocytosis and autophagy^37^. Remofuscin binds to lipofuscin^38^ and is a superoxide generator when illuminated with light^39^. Superoxide might help to degrade the polymeric lipofuscin into smaller units which then are transported out of the lysosomes by exocytosis. TFEB is an ortholog of HIH-30 in *C. elegans*, and HIH-30 is known to function as a transcription factor of *lipl-1* in *C. elegans* in a DR-like state^20^. Additionally, LIPL-1 degrades lipids in the lysosomal lipophagy process. However, although the expression of *hlh-30* was herein significantly increased at an early time point compared with that in the NC (Supplementary Fig. S4), there was no evidence of the lipophagy pathway being activated by remofuscin. Thus, the results of this study do not explain the association between lipophagy induced by remofuscin and the longevity of *C. elegans*. Therefore, further studies are needed to determine whether proton pump inhibition by remofuscin treatment contributes to extending the lifespan of *C. elegans* and whether remofuscin functions as a DR mimetic to extend the lifespan of *C. elegans*.

In conclusion, remofuscin, an inhibitor of lipofuscin accumulation, stimulates the expression of the lysosomal lipase gene *lipl-1* and the lysosomal lipid chaperone gene *lbp-8*, subsequently increasing the expression levels of the genes involved in xenobiotic detoxification through nuclear hormone receptors and thereby extending the *C. elegans* lifespan.

## Materials and methods

### Bacterial strains and culture conditions

*Escherichia coli* OP50 was obtained from the Caenorhabditis Genetics Center (CGC, USA) of the University of Minnesota and used as a food for *C. elegans. E. coli* OP50 was grown in Luria–Bertani (LB) broth (Ambrothia, Daejeon, Korea) at 37 °C overnight with shaking, collected by centrifugation at 3,000 × *g* for 10 min, washed in sterile M9 buffer, and diluted to a final concentration of 0.1 mg (wet weight) per microliter in M9 buffer^40^.

### Nematode strains and growth conditions

*C. elegans* Bristol strain N2, provided by the CGC, was used as the wild-type strain. The mutant strains VC1806 *nhr-234* (gk865), VC4077 *lbp-8* (gk5151[loxP + myo-2p::GFP::unc-54 3' UTR + rps-27p::neoR::unc-54 3' UTR + loxP]), VC875 *cyp-35A1* (ok1414), RB2046 *cyp-35A3* (ok2709), and RB2063 *gst-5* (ok2726) were provided by the CGC, and FX1954 *lipl-1* (tm1954), FX1290 *nhr-210* (tm1290), FX30306 *nhr-49* (tm7967), FX19275 *nhr-8* (tm1800), FX01722 *ahr-1* (tm1722), FX4598 *pha-4* (tm4598), FX21842 *cyp-35A2* (tm11844), and FX22344 *cyp-35A5* (tm12345) were provided by the National Bioresource Project (NBRP) for nematodes at Tokyo Women's Medical University (Tokyo, Japan). Worms were maintained and propagated in peptone-free modified nematode growth medium (mNGM) to prevent negative influence of metabolites produced by proliferated food bacteria on nematodes at 25 °C according to previously reported techniques^41,42^. *E. coli* OP50 was spread on mNGM in 90-mm-diameter Petri dishes as food for the worms. A sodium hypochlorite-sodium hydroxide solution (Sigma Aldrich, St. Louis, MO, USA) was used to obtain viable eggs as previously described^43^. The eggs were transferred onto fresh mNGM plates seeded with *E. coli* OP50 and incubated at 25 °C until the L4 stage (3-day-old worms), and all experiments used the L4 stage (3-day-old worms) as day 1 of the adult stage to control the reproductive system in *C. elegans*^42^.

### Assay of the *C. elegans* mean lifespan

Remofuscin (kindly provided by Professor Ulrich Schraermeyer, Universität Tübingen, Tübingen, Germany) was dissolved in dimethyl sulfoxide (DMSO, Sigma Aldrich) and administered final concentrations of 0 µM (control), 50 µM, 100 µM, and 200 µM. An equal amount of DMSO (final concentration, 0.2%) was added as the control. 5-Fluoro-2ʹ-deoxyuridine (FUdR, Sigma Aldrich) (50 μM) was added to the plates^44^, which were then seeded with *E. coli* OP50. The *C. elegans* mean lifespan (MLS) assay was conducted by transferring 15 young adult (L4 stage) worms onto mNGM/FUdR plates containing *E. coli* OP50 and treated with remofuscin at the indicated concentrations. For each assay, 45 worms were assayed on three plates (15 worms per plate) for each remofuscin concentration. The longevity assay was conducted three times independently at least in triplicate, and more than 100 worms were scored. The data from independent three replicates were merged and analyzed. The plates were incubated at 25 °C, and the live and dead worms were counted every 24 h. Worms were considered “dead” when they did not respond to a gentle touch with a worm picker. Nematodes that crawled off the plates and died in a non-natural manner, such as by bagging or adhering to the plate wall, were not included in the analysis (censored)^45^. The worms were transferred every two days to maintain a sufficient food source.

The MLS was estimated using the following Eq. ^46^:

1 $$\mathrm{MLS }= \frac{1}{N}\sum_{j}\frac{{\mathrm{x}}_{j}+{\mathrm{x}}_{j+1}}{2}{d}_{j}$$

In the equation, *j* is the age (day),${d}_{j}$ is the number of worms that died during the day interval (${\mathrm{x}}_{j}$, ${\mathrm{x}}_{j+1}$), and *N* is the total number of worms. The standard error (SE) of the estimated MLS was calculated using the following formula.

2 $$\mathrm{SE }= \sqrt{\frac{1}{N(N-1)}{\left(\sum_{j}\frac{{\mathrm{x}}_{j}+{\mathrm{x}}_{j+1}}{2}-\mathrm{MLS}\right)}^{2}{d}_{j}}$$

### Measurement of body length

Worms at the L4 stage (day 1 of the adult stage) were transferred onto mNGM plates (60 mm Petri dish) containing various concentrations of remofuscin and seeded with 5 mg (wet weight) of *E. coli* OP50 in M9 buffer. The plates were incubated at 25 °C, and the body lengths of live worms were measured every 24 h until 6 days of age. In total, 10 worms per group were measured. *C. elegans* were imaged with a stereomicroscope (Olympus SZ61, Tokyo, Japan) and a ToupCam (UCMOS05100KPA, ToupTek, Hangzhou, China), and the images were analyzed by using ToupCam software. The area of the worm’s projection was estimated automatically and used as an index of body length. Three independent experiments were conducted for each group.

### Measurement of the pharyngeal pumping rate

A pharyngeal pumping rate assay was performed on mNGM plates seeded with *E. coli* OP50 and treated with various concentrations of remofuscin. Three-day-old worms (L4 stage) were transferred onto mNGM plates containing various concentrations of remofuscin and incubated at 25 °C, and the number of contractions in the terminal bulb of the pharynx was counted every 48 h for 1 min using an Olympus CKX41 inverted microscope (400 ×). Three independent experiments were conducted, and 15 worms were included in each group for each measurement.

### Measurement of lipofuscin accumulation

The autofluorescence of lipofuscin in 14-day-old adult *C. elegans* was measured as an aging index. Randomly selected worms from each group were placed onto 5% agar pads coated with 10 mM sodium azide (Junsei Chemical, Tokyo, Japan) in M9 buffer for anesthetization. Images of lipofuscin autofluorescence at a blue excitation wavelength (405–488 nm), which captures 4',6-diamidino-2-phenylindole (DAPI), were acquired with a laser confocal scanning microscope (Olympus Ix81-FV1000)^40^. Fluorescence was quantified using FV10-ASW1.1 software (Olympus) to measure lipofuscin accumulation. Three independent experiments were conducted, and 10 worms were included in each group for each measurement.

### Measurement of ROS

The ROS levels in *C. elegans* treated with 0 µM, 50 µM, 100 µM, and 200 µM remofuscin were measured for 14 days. Randomly selected worms from each group were washed twice with M9 buffer, after which the supernatant was removed, and the remaining worm pellet was suspended in 100 µL of M9 buffer. The worm pellet (100 µL) and 100 µL of 50 mM 2′,7′-dichlorofluorescein diacetate (H_2-_DCF-DA, Sigma Aldrich) were added to the wells of a black 96-well plate. The ROS levels were measured with a fluorescence microplate reader (SpectraMAX GEMINI EM, Molecular Devices, Sunnyvale, CA, USA**)** at excitation and emission wavelengths of 485 nm and 520 nm, respectively, at 90 min after activation. The fluorescence signal in each group, which included more than 80 worms for each measurement, was normalized to the protein concentration in each group. Three independent experiments were conducted.

### Microarray analysis

Worms fed *E. coli* OP50 for 14 days on NGM plates containing 0 µM and 200 µM remofuscin were collected and washed twice with M9 buffer, after which total RNA was isolated from whole worms using TRIzol (Invitrogen, Carlsbad, CA, USA) according to a previously described method^13^. For each RNA, the synthesis of target cRNA probes and hybridization were performed using an Aligent LowInput QuickAmp labeling kit (Agilent Technologies, Santa Clara, CA, USA) according to the manufacturer’s instructions. Amplified and labeled cRNA was purified on a cRNA Cleanup Module (Agilent Technologies), and labeled cRNA targets were quantified using an ND-1000 spectrophotometer (NanoDrop Technologies, Inc., Wilmington, DE, USA). After checking the labeling efficiency, the cRNA was fragmented by adding 10X blocking agent and 25X fragmentation buffer and incubating at 60 °C for 30 min. The fragmented cRNA was resuspended in 2X hybridization buffer and directly pipetted onto assembled *C. elegans* oligo microarrays (Agilent, 44 K). The arrays were hybridized at 65 °C for 17 h using a hybridization oven (Agilent Technologies). The hybridized microarrays were washed according to the manufacturer’s protocol (Agilent Technologies). The hybridized images were scanned using an Agilent DNA microarray scanner and quantified with Feature Extraction 10.7 software (Agilent Technologies). Raw intensity data were globally normalized^47^. All data normalization and selection of differentially expressed genes (fold change) were performed using GeneSpring GX 7.3.1 (Agilent Technologies). The criterion for the identification of genes with significantly altered expression was a *p* value < 0.05 compared with the negative control. The RNA sequencing data were deposited in the NCBI Gene Expression Omnibus (GEO) database (accession code: GSE144059). For the pie chart and volcano plot, Excel-based Differentially Expressed Gene Analysis (ExDEGA, eBiogen, Seoul, Korea) software was used to analyze the microarray data according to classified Gene Ontology (GO) terms. Genes with a *p*-value < 0.05 and a fold change of 2.0 compared with the negative control were defined as significantly changed genes.

### Quantitative real-time polymerase chain reaction (qPCR)

*C. elegans* fed *E. coli* OP50 for 1 and 5 days on NGM plates containing 0 µM and 200 µM remofuscin were collected and washed twice with M9 buffer. Total mRNA was isolated from whole worms using TRIzol (Invitrogen) as previously described^13^. The RNA was converted into cDNA using a RevertAid First Strand cDNA Synthesis Kit according to the manufacturer’s instructions (Thermo Scientific, Wilmington, DE, USA) and then amplified by qPCR using SYBR Green (KAPA Biosystems, Wilmington, MA, USA) and a QuantStudio 6 Flex Real Time PCR machine (Applied Biosystems, Foster City, CA, USA). For qPCR, an initial step at 95 °C followed by 40 cycles of 95 °C for 15 s, 60 °C for 15 s, and 72 °C for 30 s were performed, and melting curve analysis was performed. The experiments were independently conducted at least three times, and relative expression levels were calculated using the 2^−ΔΔCT^ method^48^. The internal control gene *act-1* was used to normalize the gene expression data. The sequences of the primers used in this study are listed in Supplementary Table S3.

### Statistical analysis

In the lifespan assay, the Kaplan–Meier method and the log-rank test were used to calculate the MLS and *p*-values, respectively^40^. In the other experiments, the significance of comparisons between the negative control (NC) and remofuscin-treated groups was calculated by using Student’s *t*-test. Significance was defined as a *p*-value less than 0.05 in all experiments. If the data were not normally distributed, the Mann–Whitney U test was used^42^.

## Supplementary Information

Supplementary Information 1.

Supplementary Information 2.
