# Supplementary material for: Remofuscin induces xenobiotic detoxification via a lysosome-to-nucleus signaling pathway to extend the Caenorhabditis elegans lifespan
Source: Sci Rep. 2022 May 3;12:7161. doi: 10.1038/s41598-022-11325-2 (PMC9064964; doi:10.1038/s41598-022-11325-2)
Supplement: Supplementary file 2 — Supplementary Information 2. [file 41598_2022_11325_MOESM2_ESM.docx]

**Supplementary Information**

**Remofuscin induces xenobiotic detoxification via a lysosome-to-nucleus signaling pathway to extend the *Caenorhabditis* *elegans* lifespan**

**Authors: Miae Oh, Jiah Yeom, Ulrich Schraermeyer, Sylvie Julien-Schraermeyer, Young-Hee Lim***

**Table S1.** Xenobiotic metabolism-related genes upregulated in *C. elegans* (N2) treated with 200 μM remofuscin analyzed by RNA microarray.

| Gene name | Wormbase ID | Brief description | Fold change | *p*-value |
| --- | --- | --- | --- | --- |
| *cyp-13A2* | WBGene00011676 | Phase Ⅰ detoxification, cytochrome P450 | 2.387 | 0.018 |
| *cyp-34A1* | WBGene00011698 | Phase Ⅰ detoxification, cytochrome P450 | 5.424 | 0.007 |
| *cyp-35A1* | WBGene00015399 | Phase Ⅰ detoxification, cytochrome P450 | 18.131 | 0.014 |
| *cyp-35A2* | WBGene00015400 | Phase Ⅰ detoxification, cytochrome P450 | 8.568 | 0.007 |
| *cyp-35A3* | WBGene00019565 | Phase Ⅰ detoxification, cytochrome P450 | 110.931 | 0.004 |
| *cyp-35A4* | WBGene00016786 | Phase Ⅰ detoxification, cytochrome P450 | 76.458 | 0.000 |
| *cyp-35A5* | WBGene00019473 | Phase Ⅰ detoxification, cytochrome P450 | 30.956 | 0.009 |
| *cyp-35C1* | WBGene00007362 | Phase Ⅰ detoxification, cytochrome P450 | 14.545 | 0.038 |
| *gst-28* | WBGene00001776 | Phase Ⅱ detoxification, glutathione S transferase | 3.080 | 0.033 |
| *gst-5* | WBGene00001753 | Phase Ⅱ detoxification, glutathione S transferase | 2.171 | 0.008 |
| *ugt-65* | WBGene00008583 | Phase Ⅱ detoxification, UDP-glucuronosyl transferase | 2.662 | 0.026 |
| *pgp-14* | WBGene00004008 | Phase Ⅲ detoxification, a member of the ABC transporter family | 2.299 | 0.022 |
| *folt-2* | WBGene00018138 | Phase Ⅲ detoxification, folate transmembrane transporter | 2.821 | 0.022 |

Fold changes were calculated between the control and remofuscin (200 μM)-treated *C. elegans*.

**Table S2.** List of nuclear hormone receptor genes upregulated in *C. elegans* (N2) treated with 200 μM remofuscin as determined by microarray.

| Gene name | Wormbase ID | Brief descriptions | Fold change | *p*-value |
| --- | --- | --- | --- | --- |
| Lysosome | | | | |
| *lipl-1* | WBGene00010062 | Lysosomal lipase | 2.635 | 0.010 |
| *lbp-8* | WBGene00002260 | Lipid binding protein | 3.200 | 0.031 |
| Nuclear hormone receptor | | | | |
| *nhr-184* | WBGene00018415 | Nuclear hormone receptor family | 2.026 | 0.089 |
| *nhr-210* | WBGene00020015 | Nuclear hormone receptor family | 2.385 | 0.204 |
| *nhr-234* | WBGene00012596 | Nuclear hormone receptor family | 2.249 | 0.400 |
| *nhr-265* | WBGene00009608 | Nuclear hormone receptor family | 2.042 | 0.103 |
| *nhr-34* | WBGene00003627 | Nuclear hormone receptor family | 2.509 | 0.278 |
| *nhr-38* | WBGene00003629 | Nuclear hormone receptor family | 2.714 | 0.268 |
| Fatty acid beta-oxidation | | | | |
| *ech-9* | WBGene00001158 | Enoyl-CoA hydratase | 1.729 | 0.199 |

Fold changes were calculated between the control and the remofuscin (200 μM)-treated *C. elegans*.

**Table S3.** Primer sequences used in this study.

| Gene |  | Primer sequences |
| --- | --- | --- |
| *act-1* | Forward | 5' CACGGTATCGTCACCAACTG 3' |
|  | Reverse | 5' GCTTCAGTGAGGAGGACTGG 3' |
| *lipl-1* | Forward | 5' CGGTTTGCGCTGGACTTA 3' |
|  | Reverse | 5' GAACACGAGTTGCGTTAA 3' |
| *lbp-8* | Forward | 5' AATTGCTCCGGATGAGCGATCCTA 3' |
|  | Reverse | 5' TCTCTACGACAAATGACGCTCCCA 3' |
| *nhr-210* | Forward | 5' CTGAAAGCTCGTCAGTCTTC 3' |
|  | Reverse | 5' GTAGCTTCATCCAGCAGGTA 3' |
| *nhr-234* | Forward | 5' GTGCCGATACTCCAACAA 3' |
|  | Reverse | 5' TGTATCCACGGACTACTTCC 3' |
| *nhr-49* | Forward | 5' TCACCTCATCTCATCTCCAG 3' |
|  | Reverse | 5' AGAGATGGCCTAGACAGGTT 3' |
| *acox-3* | Forward | 5' ATCCCAGTTCTCGAGTATCC 3' |
|  | Reverse | 5' GGAGAGCTCGTCTGACTTTT 3' |
| *ech-9* | Forward | 5' CTCTGCCAAATGATCGAG 3' |
|  | Reverse | 5' CTTGCTTGCATCTCTGCT 3' |
| *nhr-8* | Forward | 5' CAACAGCTGTGAACAGTCC 3' |
|  | Reverse | 5' CGGAGAAACTGTGTAGTTGG 3' |
| *ahr-1* | Forward | 5' AGCCGTCTGGATAAACTCTC 3' |
|  | Reverse | 5' ATGGTATAGGAGGGTGTGGT3' |
| *pha-4* | Forward | 5' GCAAGCACAGATGACACTC 3' |
|  | Reverse | 5' CTGGTATACTCCGTTGGTGT 3' |
| *cyp-34A1* | Forward | 5' GTTGATGGTCAACCTCTACG 3' |
|  | Reverse | 5' AGCCTTGGCTAGTGATTCTC 3' |
| *cyp-35A1* | Forward | 5' ATACTACCTGTGGGCAACTG 3' |
|  | Reverse | 5' CGTGAGCTATATCGGCATAC 3' |
| *cyp-35A2* | Forward | 5' CTCAACTCAGTGCTCTCCAT 3' |
|  | Reverse | 5' GAAGGAGAAGGTTACCGAAG 3' |
| *cyp-35A3* | Forward | 5' TGGTTCTCGAAGTCTCAGTC 3' |
|  | Reverse | 5' GAGCAGTAACAAGAGCTCCA 3' |
| *cyp-35A4* | Forward | 5' CTTACTGACCGTGCTTCAAC 3' |
|  | Reverse | 5' GAGCAGTAACAAGAGCTCCA 3' |
| *cyp-35A5* | Forward | 5' GGATAAGGCAAGAGAAGAGC 3' |
|  | Reverse | 5' TGTAACGACAGCTCCAGAGT 3' |
| *cyp-35C1* | Forward | 5' CTGTCGACTCAGGATGTTTG 3' |
|  | Reverse | 5' AGATTCTCCCAGACAAGACC 3' |
| *gst-5* | Forward | 5' GCCGGACAACAATACGAGGA 3' |
|  | Reverse | 5' AAGAAACGAGCAATCGCGTG 3' |
| *gst-28* | Forward | 5' CACGTATCCTCTTCCACTTGCTG 3' |
|  | Reverse | 5' CTGCTCTTCTGGGGTTTT 3' |
| *hlh-30* | Forward | 5' AGTAGCAGCAAGTCACCATC 3' |
|  | Reverse | 5' GATCAGGTCGTCAAGCTCT 3' |

**Fig. S1.** Effect of remofuscin on the lifespan of *C. elegans* (N2). Worms at the L4 stage (day 1 of the adult stage) fed on the *E. coli* OP50 lawn were treated with various concentrations of remofuscin. The survival percentages were calculated using the Kaplan-Meier method. ^*^*p* < 0.05, ^***^*p* < 0.001, log-rank test, compared with the NC.

**Fig. S2.** Expression levels of genes related to transcription factors in remofuscin-treated *C. elegans*. Worms were grown on NGM plates containing 0 µM and 200 µM remofuscin for 5 days. The gene expression levels were measured by qPCR.


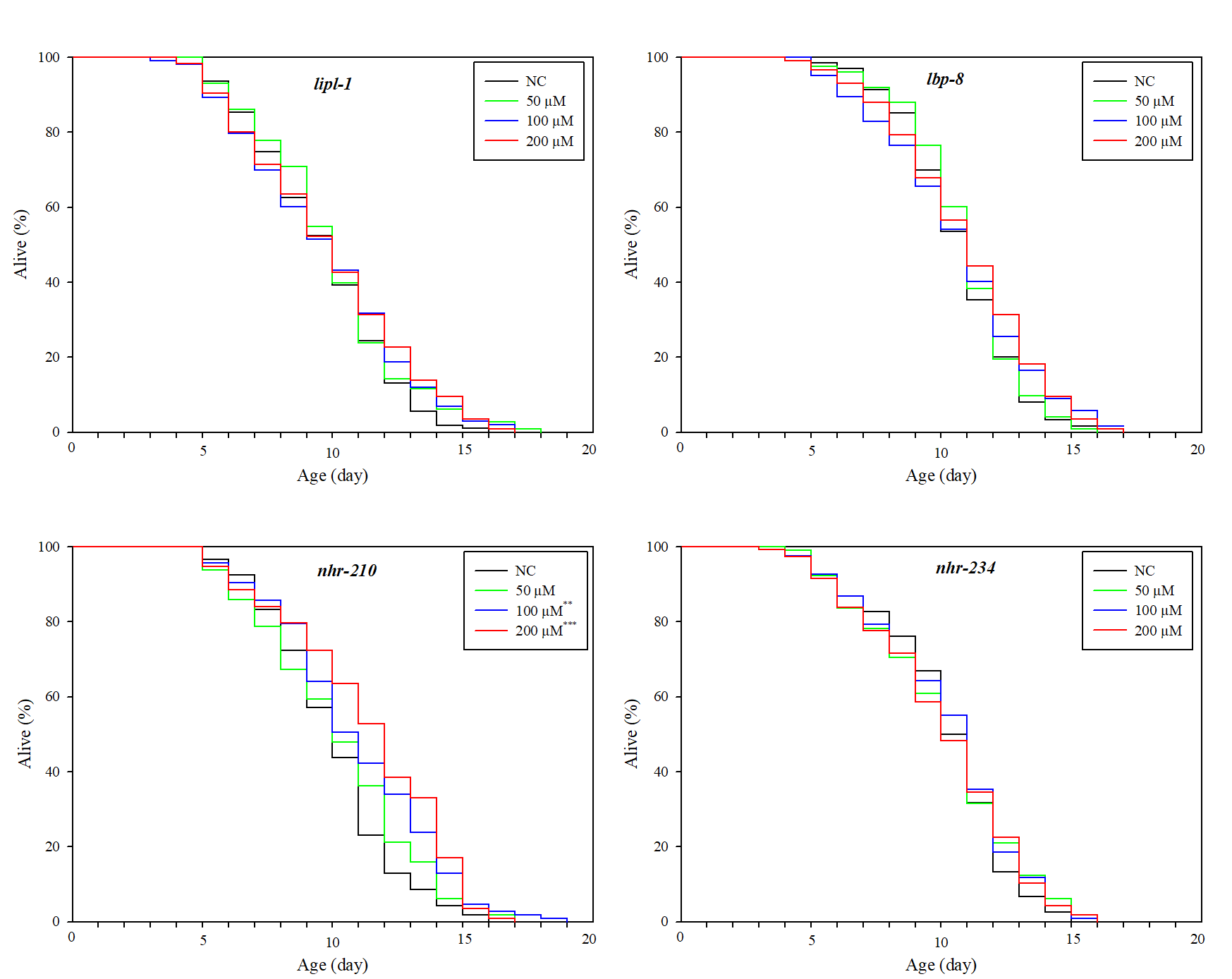


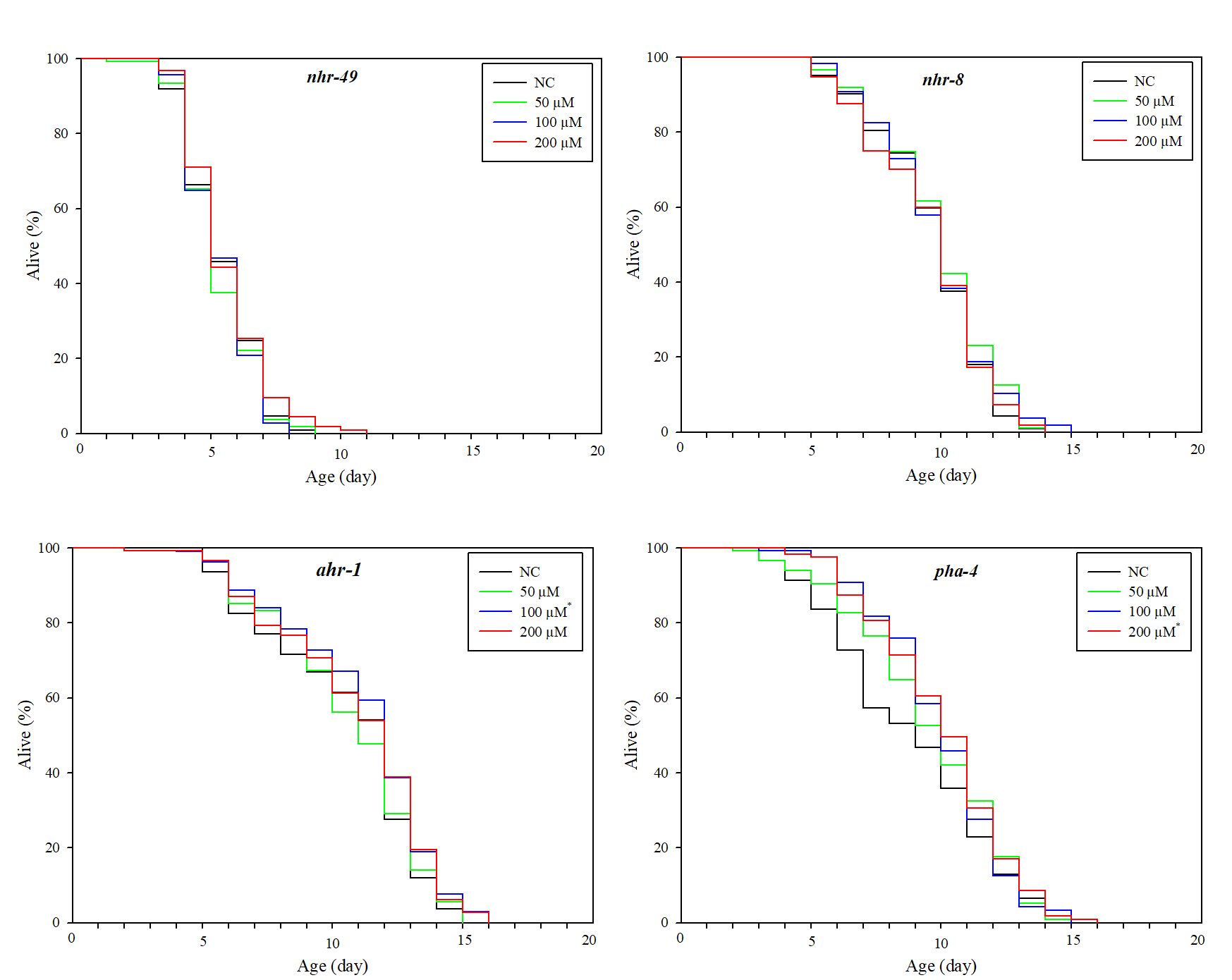


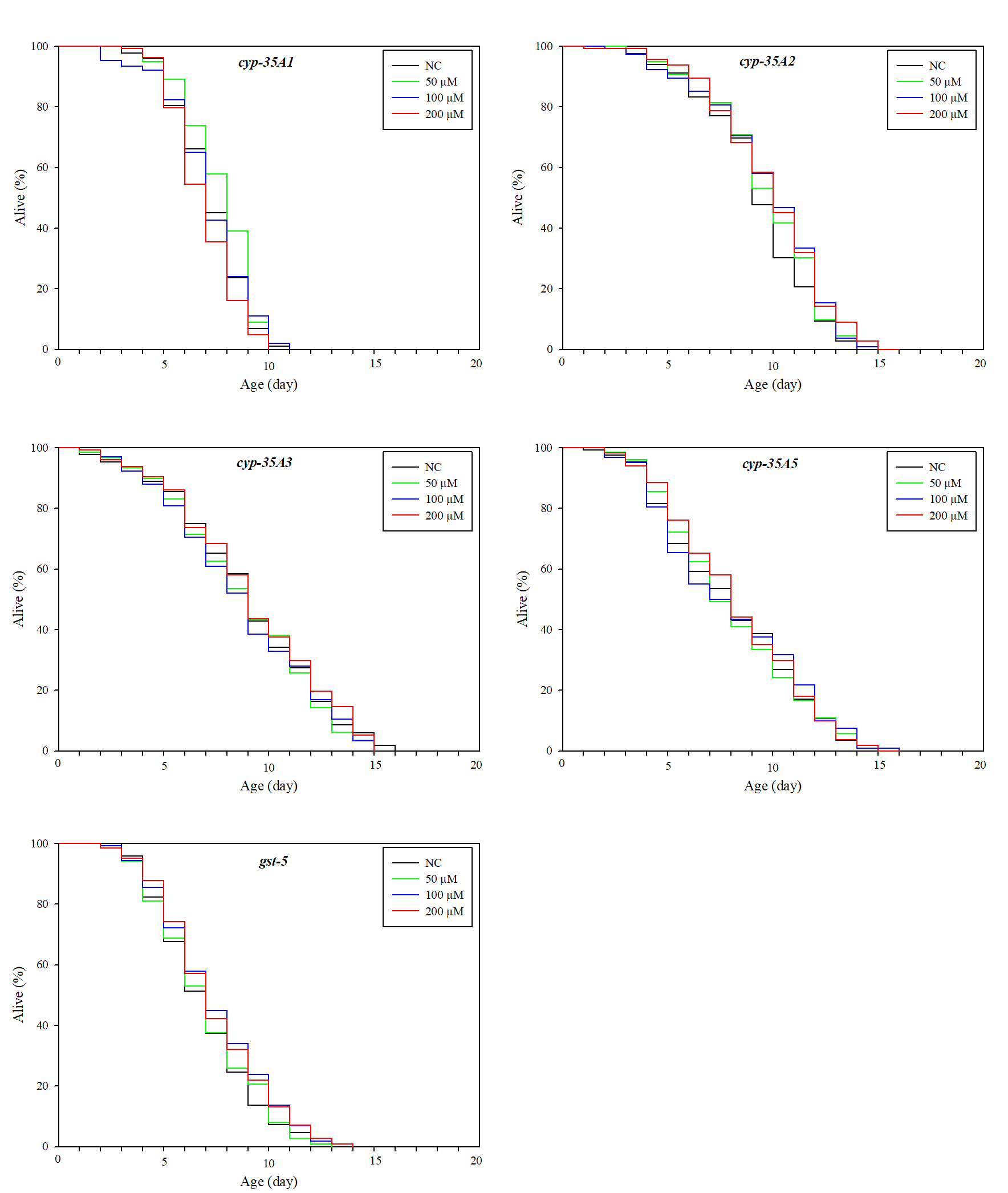


**Fig. S3.** Effect of remofuscin on the lifespan extension of *C. elegans* loss-of-function mutants. ^*^*p* < 0.05, ^**^*p* < 0.01, ^***^*p* < 0.001, log-rank test, compared with the NC (0 μM remofuscin).

**Fig. S4.** Expression levels of *hlh-30* in remofuscin-treated *C. elegans*. Worms were grown on NGM plates containing 0 µM and 200 µM remofuscin for 1 and 5 days. The gene expression levels were measured by qPCR.
